# Supplementary material for: Pharmacological blockade of TEAD–YAP reveals its therapeutic limitation in cancer cells
Source: Nat Commun. 2022 Nov 8;13:6744. doi: 10.1038/s41467-022-34559-0 (PMC9643419; doi:10.1038/s41467-022-34559-0)
Supplement: Supplementary file 1 — Supplementary Information [file 41467_2022_34559_MOESM1_ESM.pdf]

Supplementary Information

Pharmacological blockade of TEAD–YAP reveals its therapeutic limitation in cancer cells

Yang Sun[1,6,7,*], Lu Hu[1,6], Zhipeng Tao[1,6], Gopala K Jarugumilli[1], Hannah Erb[1], Alka Singh[2], Qi Li[2], Jennifer L. Cotton[2], Patricia Greninger[3], Regina K. Egan[3], Y. Tony Ip[4], Cyril H. Benes[3], Jianwei Che[5], Junhao Mao[2,*], Xu Wu[1,*]

[1] Cutaneous Biology Research Center, Massachusetts General Hospital, Harvard Medical School, Charlestown, Massachusetts, USA.

[2] Department of Molecular, Cell and Cancer Biology, University of Massachusetts Chan Medical School, Worcester, Massachusetts, USA.

[3] Massachusetts General Hospital Cancer Center, and Department of Medicine, Harvard Medical School, Charlestown, Massachusetts, USA.

[4] Program in Molecular Medicine, University of Massachusetts Chan Medical School, Worcester, Massachusetts, USA.

[5] Department of Cancer Biology, Dana Farber Cancer Institute, and Harvard Medical School, Boston, Massachusetts, USA

[6] These authors contributed equally to this work.

[7] Present Address: Cancer Institute, Xuzhou Medical University, Xuzhou, Jiangsu, China.

*Corresponding authors

xwu@cbrc2.mgh.harvard.edu (X.W)

Junhao.Mao@umassmed.edu (J.M)

yangsun@xzhmu.edu.cn (Y.S)

Contains:

Supplementary Fig. 1-16
Supplementary Table 1-3

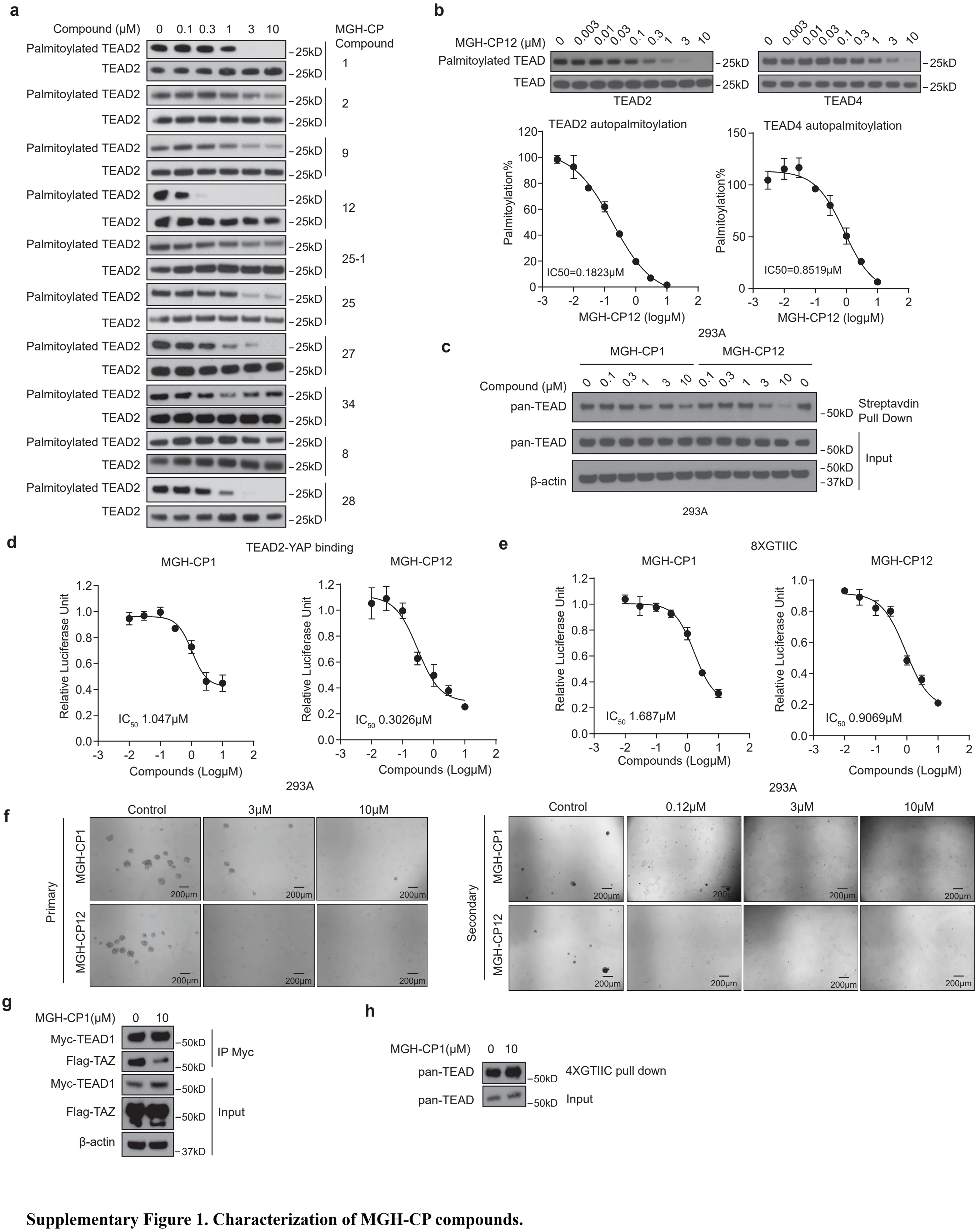

Supplementary Figure 1. Characterization of MGH-CP compounds.

a. Autopalmitoylation of recombinant TEAD2 (YAP binding domain, YBD) with MGH-CP compounds with 1μM alkyne palmitoyl-CoA in vitro. Palmitoylation of TEAD2 indicates protein labeled with alkyne palmitoyl-CoA assessed by adding biotin tag using click chemistry. TEAD2 indicates the total loading protein in the assay (three experiments were repeated independently with similar results). **b**. Autopalmitoylation assay of recombinant TEAD2 and TEAD4 protein (YAP binding domain, YBD) with MGH-CP12 at different concentrations. The IC50s of autopalmitoylation inhibition were shown (n=3 biological repeats). **c**. Pan-TEAD palmitoylation inhibition was shown in HEK293A cells treated with MGH-CP1 or MGH-CP12. Metabolic labeling with a chemical reporter of palmitoylation (Alk-C16) was used to determine TEAD palmitoylation levels (Three experiments were repeated independently with similar results). **d**. Inhibition of TEAD2 and YAP binding in Gal4-TEAD2-YAP binding reporter assay by MGH-CP1 and MGH-CP12 at difference concentrations (n=3 biological repeats). **e**. MGH-CP1 and CP12 inhibit TEAD-binding element–driven luciferase reporter (8xGTIIC-luciferase) at different concentrations (n=3 biological repeats). **f**. Representative images of primary and secondary Huh7 tumor spheres treated with MGH-CP1 and MGH-CP12 with indicated concentrations. Scale bar, 200μm. **g**. Co-immunoprecipitation assay was utilized to examine the myc-TEAD1 and Flag-TAZ binding in the presence of MGH-CP1. **h**. 4XGTIIC DNA (biotin labeled) pull-down assay shows that MGH-CP1 does not block TEAD-DNA binding. (**f-h**) three experiments were repeated independently with similar results. Data are represented as mean ± S.E.M. Source data are provided as a Source Data file.

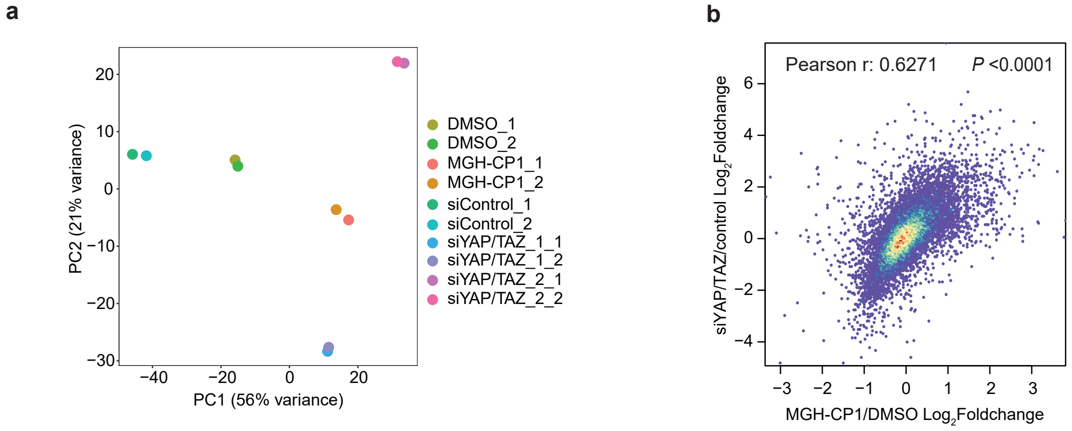

Supplementary Figure 2. Bioinformatic analysis of global genomic alterations with treatment of MGH-CP1 or knockdown of YAP/TAZ

a. Principle component analysis (PCA) of RNA-seq datasets of MGH-CP1 treatment and YAP/TAZ knockdown in MDA-MB-231 cells. **b**. Pearson correlation analysis of gene transcriptional fold change between MGH-CP1 treatment and siYAP/TAZ in MDA-MB-231 cells. Two-tailed Pearson correlation analysis were used, Pearson r 95% confidence interval 0.6164 to 0.6375, $R^2 = 0.3932$.

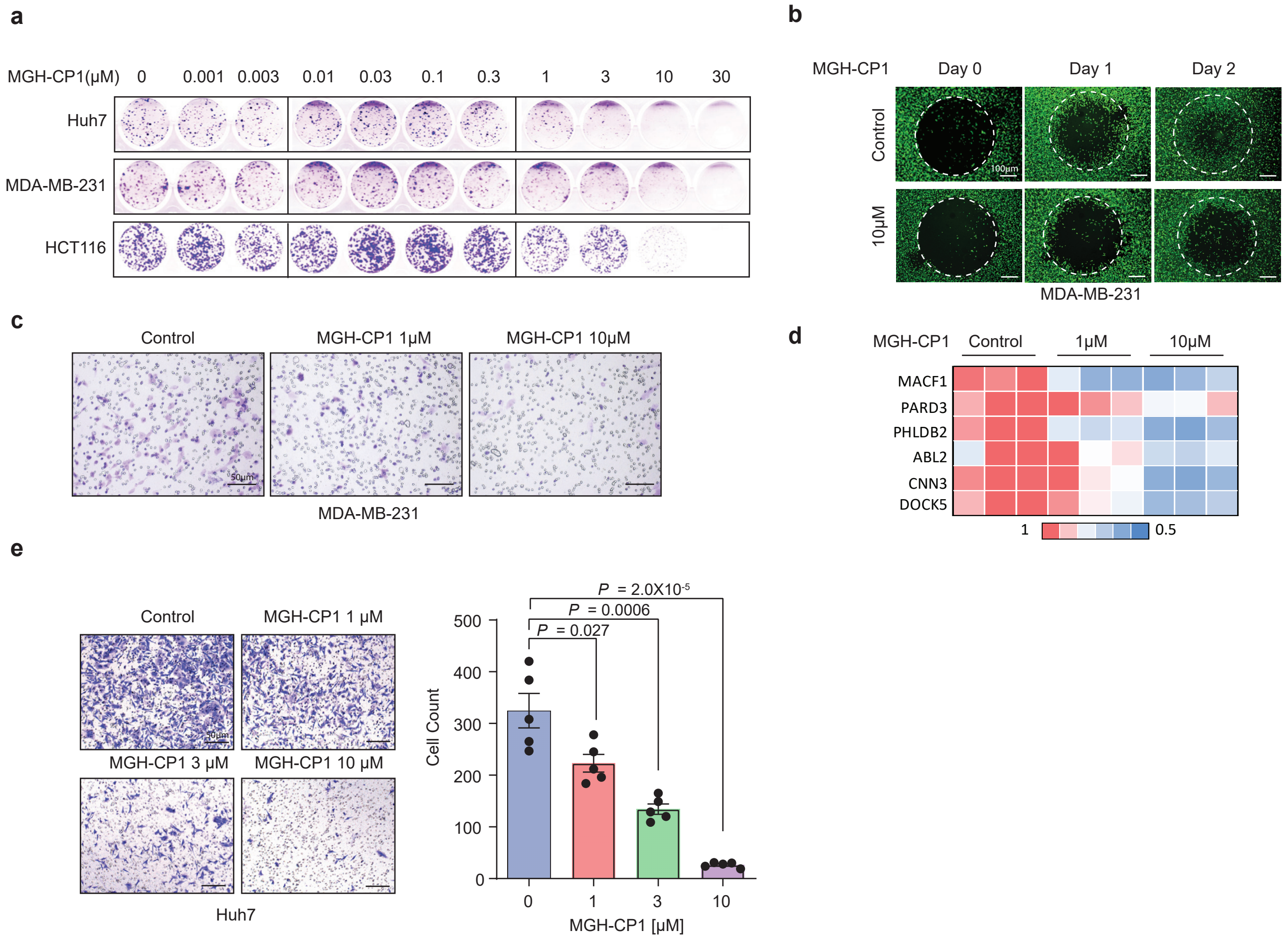

Supplementary Figure 3. MGH-CP1 inhibits tumor growth and migration.
a. MGH-CP1 blocks anchorage-independent growth of Huh7, MDA-MB-231 and HCT116 tumor cells in a dose-dependent manner. **b**. Wound healing assay shows MGH-CP1 at indicated doses block MDA-MB-231 cell migration (Three experiments were repeated independently with similar results). Scale bar, 100µm. **c**. Trans-well assay shows inhibition of MDA-MB-231 cell migration by MGH-CP1(Three experiments were repeated independently with similar results). Scale bar, 50µm. **d**. Heatmap of migration related genes inhibited by MGH-CP1 at 1 and 10µM in MDA-MB-231 cells. Scale bar, 50µm. **e**. Trans-well assay shows inhibition of Huh7 cell migration by MGH-CP1. Migrated tumor cells were counted and shown (n=5 biological repeats). Scale bar, 50µm. Data are represented as mean ± S.E.M. *P* values were determined using two-tailed t-tests.. Source data are provided as a Source Data file.

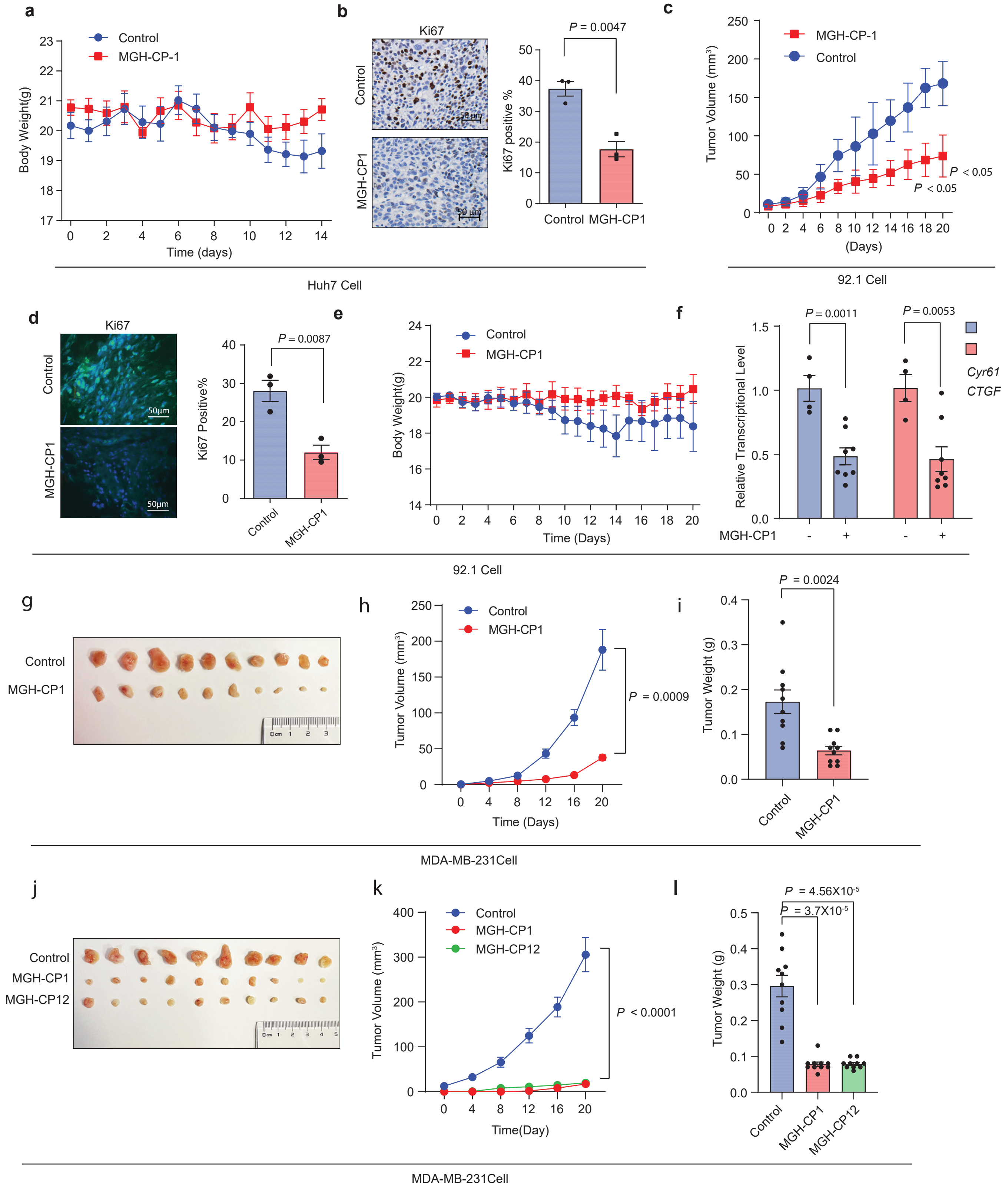

Supplementary Figure 4. MGH-CP1 attenuate tumor growth and initiation in vivo.
a. Body weight of the mice inoculated with Huh7 cells treated with vehicle control or MGH-CP1 (i.p., 50mg/kg) (n=mice). **b**. Immunohistochemistry staining of Ki67 in Huh7 xenograft tumor treated with vehicle control or MGH-CP1(i.p.,50mg/kg). The statistical analysis of Ki67 was shown (n=3 biological repeats). Scale bar, 50µm. **c**. Tumor volume of the mice inoculated uveal melanoma cell 92.1 treated with vehicle control or MGH-CP1(i.p.,50mg/kg) (n=8, 10 tumors for Control and MGH-CP1 separately). **d**. Representative images and statistical analysis of Ki67 immunofluorescent staining of 92.1 xenograft tumor treated with vehicle control or MGH-CP1(i.p.,50mg/kg) (n=3 biological repeats). Scale bar, 50µm. **e**. Body weight of the mice inoculated with 92.1 cells treated with vehicle control or MGH-CP1 (i.p., 50mg/kg) (n=5mice). **f**. Transcriptional levels of *Cyr61* and *CTGF* in 92.1 xenograft tumors treated with vehicle control or MGH-CP1 (i.p., 50mg/kg) (n=4, 8 tumors for Control and MGH-CP1 separately). **g**. MGH-CP1 inhibits MDA-MB-231 tumor initiation. Experiment was performed as treating mice with MGH-CP1 at the following day of MDA-MB-231 cells inoculation, for 2 weeks. Representative images of xenograft tumors treated with vehicle control or MGH-CP1 (75mg/kg). Tumor volumes and weight were determined (**h** and **i**) (n=10 tumors). **j**. Representative images of MDA-MB-231xenograft tumors pre-treated with DMSO, MGH-CP1 and MGH-CP12 (10uM for 48h) in vitro, before inoculation into animals. Tumor volumes and weight were determined (**k** and **l**) (n=10 tumors). Data are represented as mean ± S.E.M. *P* values were determined using two-tailed t-tests. Time courses were analyzed by repeated measurements (mixed model) ANOVA with Bonferroni post-tests. Source data are provided as a Source Data file.

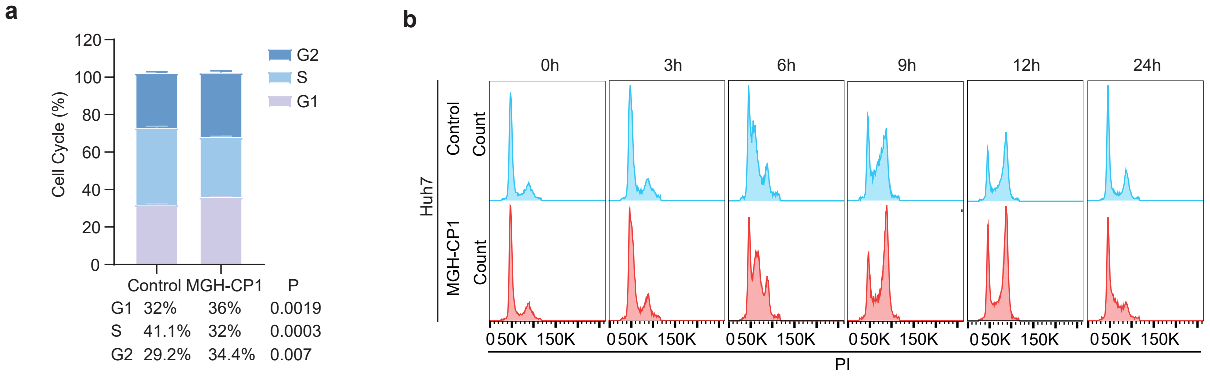

Supplementary Figure 5. TEAD autopalmitoylation inhibitor inhibits cell cycle progression.
a. Cell cycle analysis of Huh7 cells treated with MGH-CP1 at indicated time point (n=3 biological repeats). **b.** Cell cycle analysis of 10μM MGH-CP1 at indicated time point after double thymidine blocks (DTB). Data are represented as mean ± S.E.M. *P* values were determined using two-tailed t-tests. Source data are provided as a Source Data file.

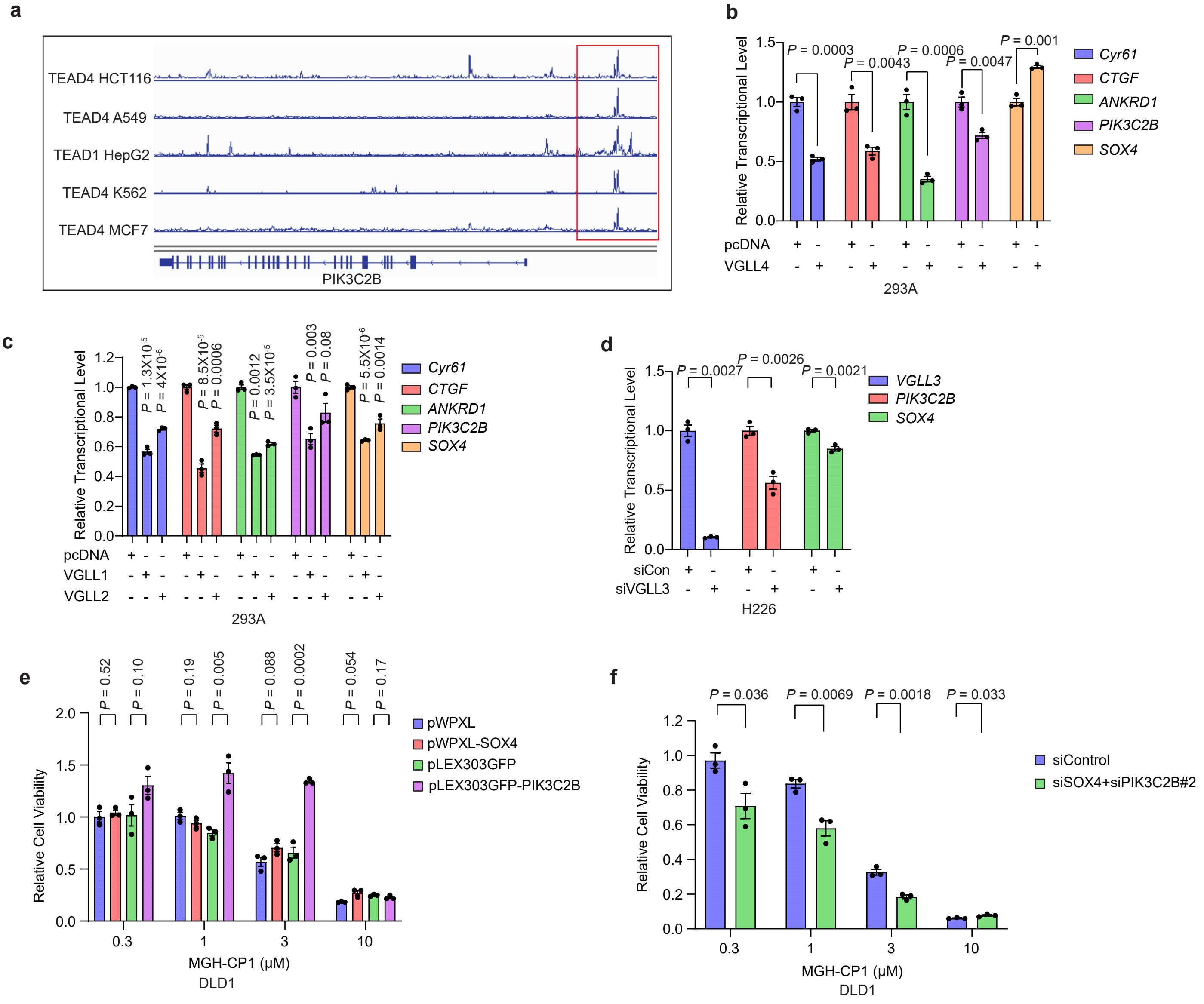

Supplementary Figure 6. VGLL3 is involved in PIK3C2B and SOX4 activation induced by TEAD inhibition.
a. Bioinformatical analysis of ChIP-seq data for TEAD at PIK3C2B regulatory region. Transcriptional levels of *Cyr61*, *CTGF*, *ANKRD1*, *PIK3C2B* and *SOX4* in 293A cells overexpressed with pcDNA or VGLL4 (**b**) or VGLL1/VGLL2(**c**) (n=3 biological repeats). **d**. VGLL3 was knocked down by siRNA in NCI-H226 cells, and *VGLL3*, *PIK3C2B* and *SOX4* transcriptional levels were determined (n=3 biological repeats). **e**. DLD1 cells were overexpressed with SOX4 or PIK3C2B in the presence of MGH-CP1. Cell viability was shown at different concentrations (n=3 biological repeats). **f**. DLD1 cells were treated with control siRNA and SOX4/PIK3C2B siRNA. Cell viability with treatment of different concentration of MGH-CP1 was determined (n=3 biological repeats). Data are represented as mean ± S.E.M. *P* values were determined using two-tailed t-tests. Source data are provided as a Source Data file.

a

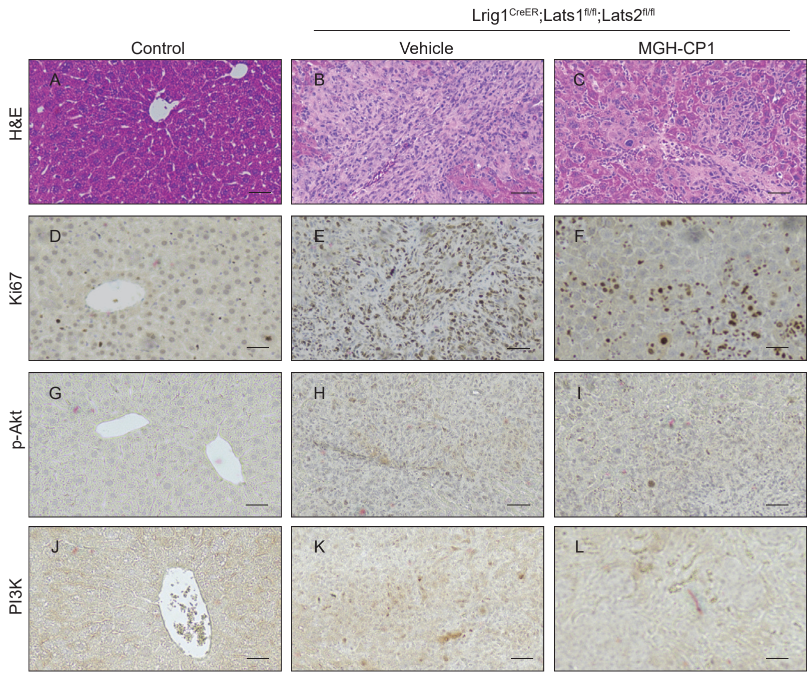

Supplementary Figure 7. The effect of MGH-CP1 treatment on AKT activation and PI3K expression in mouse liver carrying Lats1/2 deletion

a. (A-L) Histological (A-C) and immunohistochemistry (D-L) images of Ki67, phospho-AKT (p-AKT) and PI3K in the liver of control mice or Lrig1^{CreER} ; Lats1$^{fl/fl}$ Lats2$^{fl/fl}$ mice with or without MGH-CP1 treatment following Tamoxifen induction (Experiments were performed 3 independent times and representative images were shown). Scale bars, 50 µm.

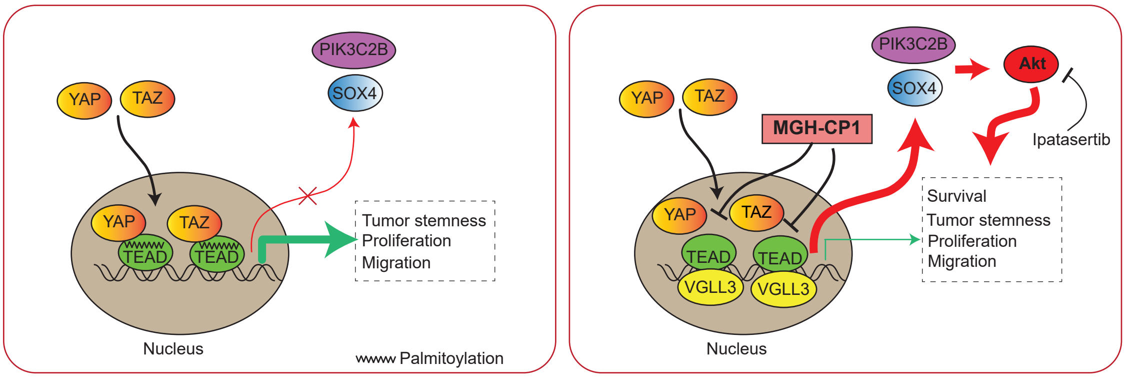

Supplementary Figure 8. Scheme of the proposed mechanism of TEAD inhibition and VGLL3-mediated PI3K/SOX4/AKT activation.

Left panel: Scheme shows that TEAD palmitoylation inhibitor (MGH-CP1) inhibits tumor cell stemness, migration and proliferation. Right panel: Inhibition of TEAD-YAP/TAZ complex leads to VGLL3-mediated transcriptional activation of PIK3C2B and SOX4, resulting in AKT activation and cell survival. Combinational treatment of TEAD and Akt inhibitors could block the feedback regulation and enhance the anti-tumor effects.

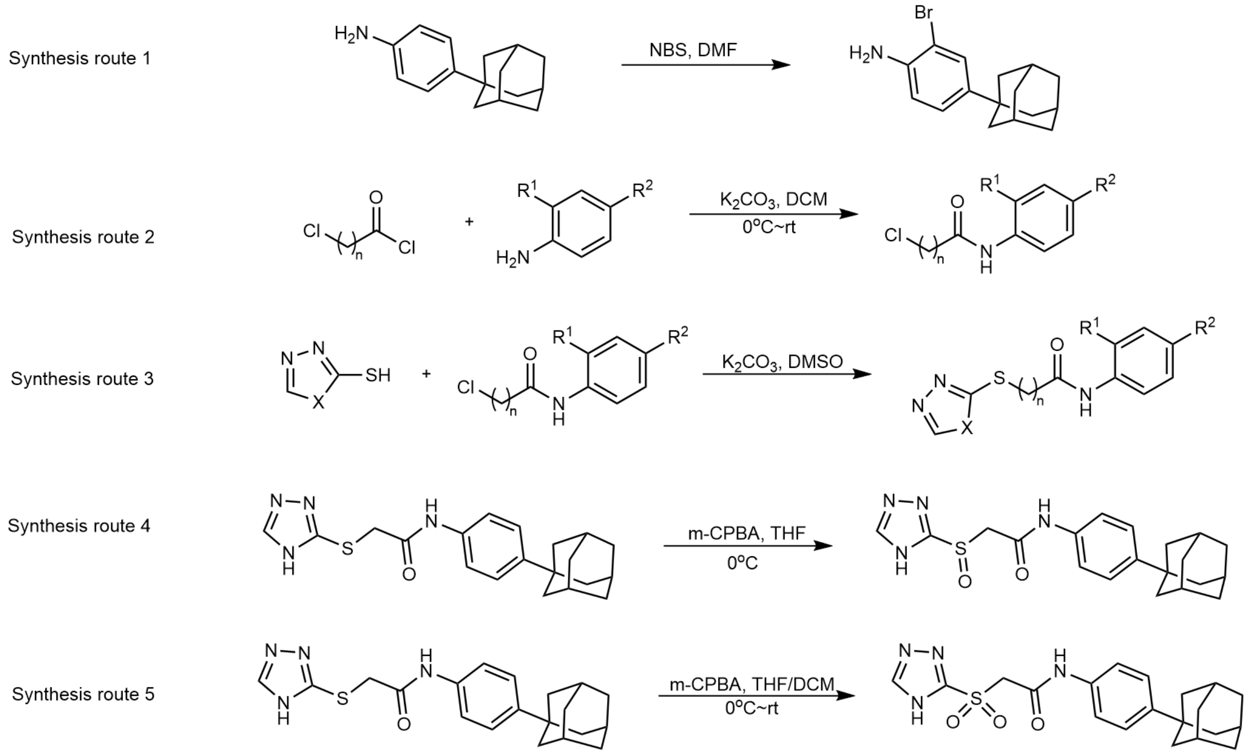

Supplementary Figure 9. MGH-CP compounds synthesis routes. Synthesis route 1 to 5 illustrating the synthetic schemes of MGH-CP compounds. See methods section for the description of the synthesis method.

Figure 1d

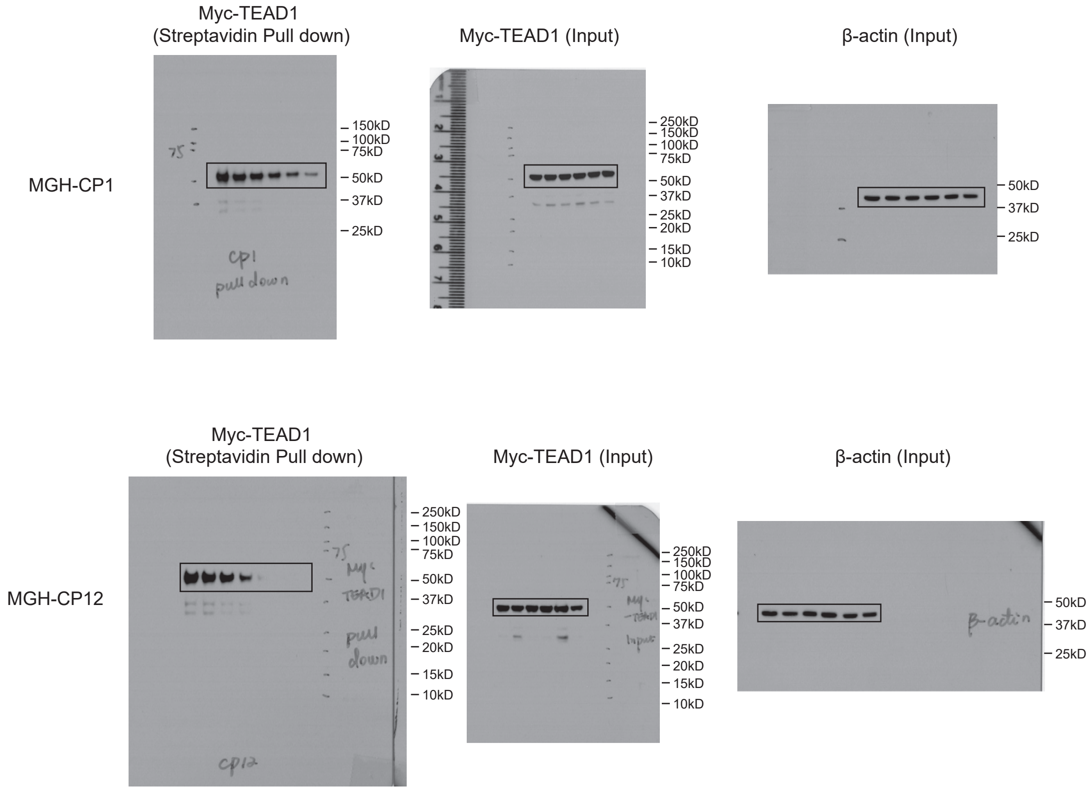

Supplementary Figure 10. Uncropped gel images for Figure 1d.

Figure 5a

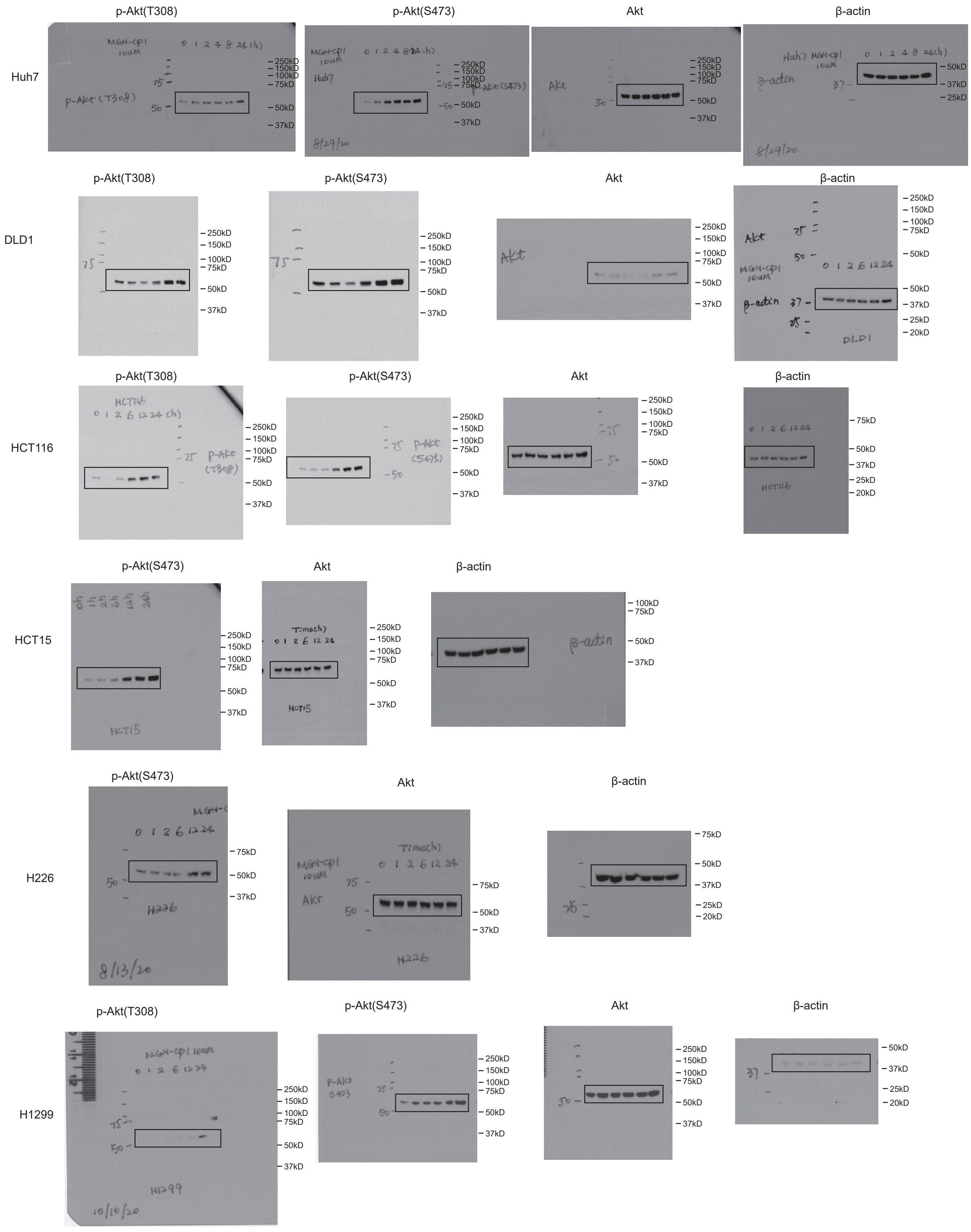

Supplementary Figure 11. Uncropped gel images for Figure 5a.

Figure 5b

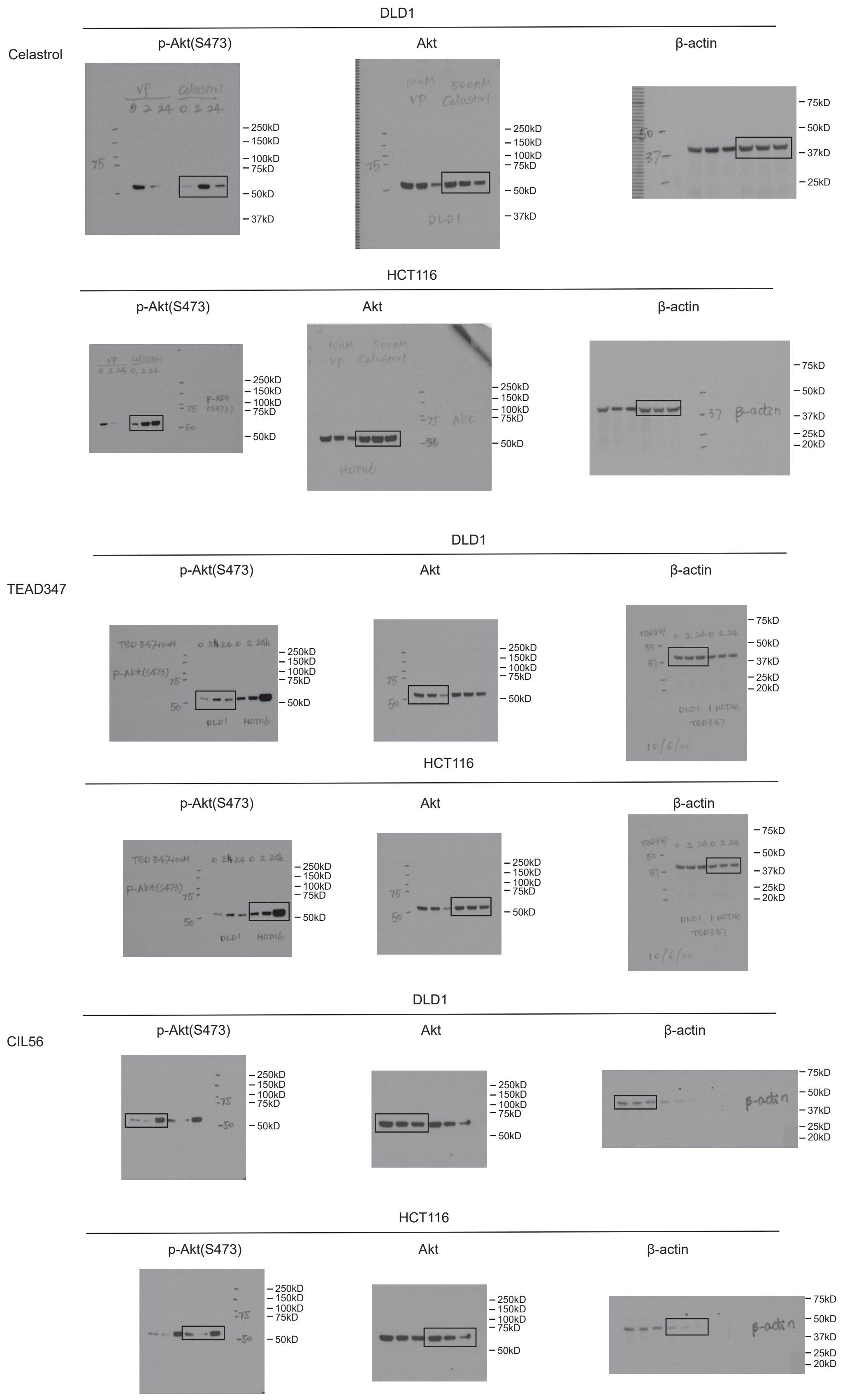

Supplementary Figure 12. Uncropped gel images for Figure 5b.

Figure 5c

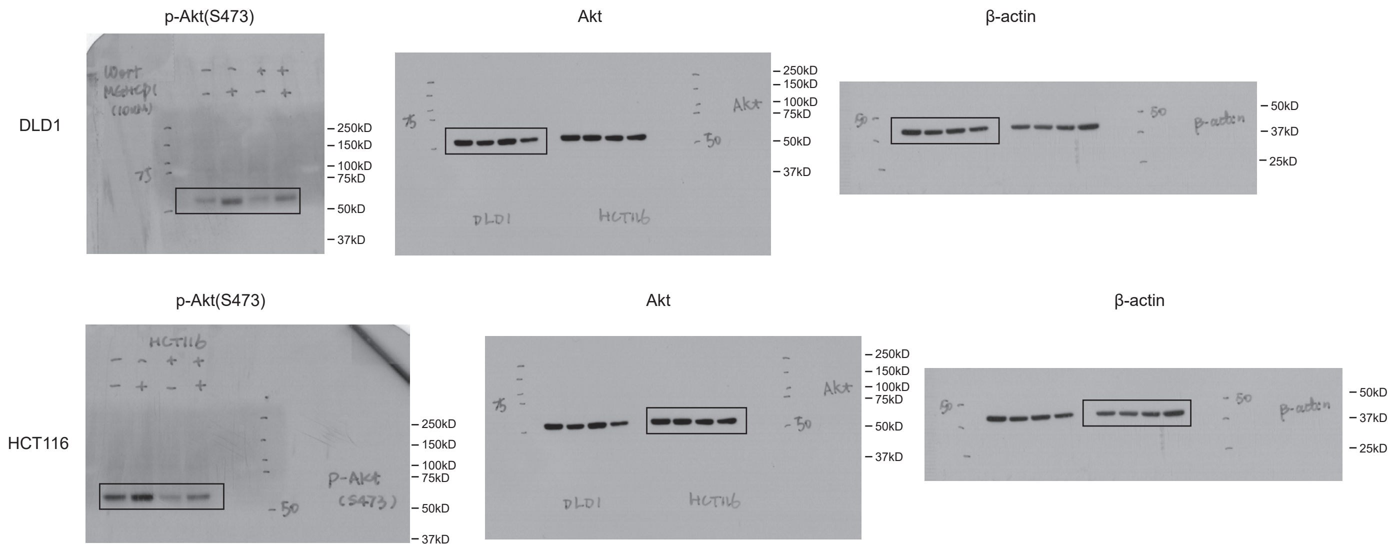

Figure 5d

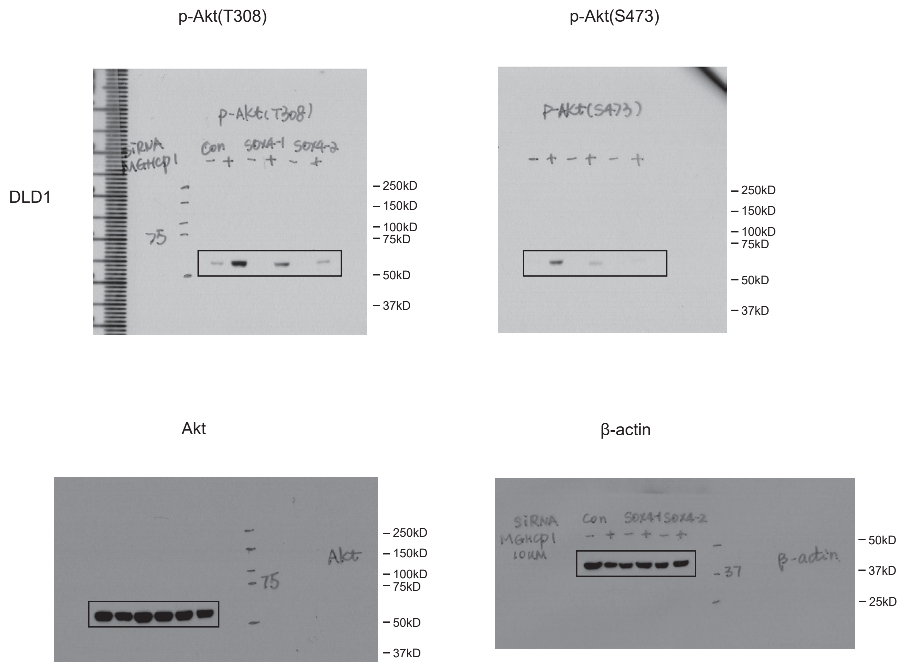

Supplementary Figure 13. Uncropped gel images for Figure 5c and d.

Supplementary Figure 1a

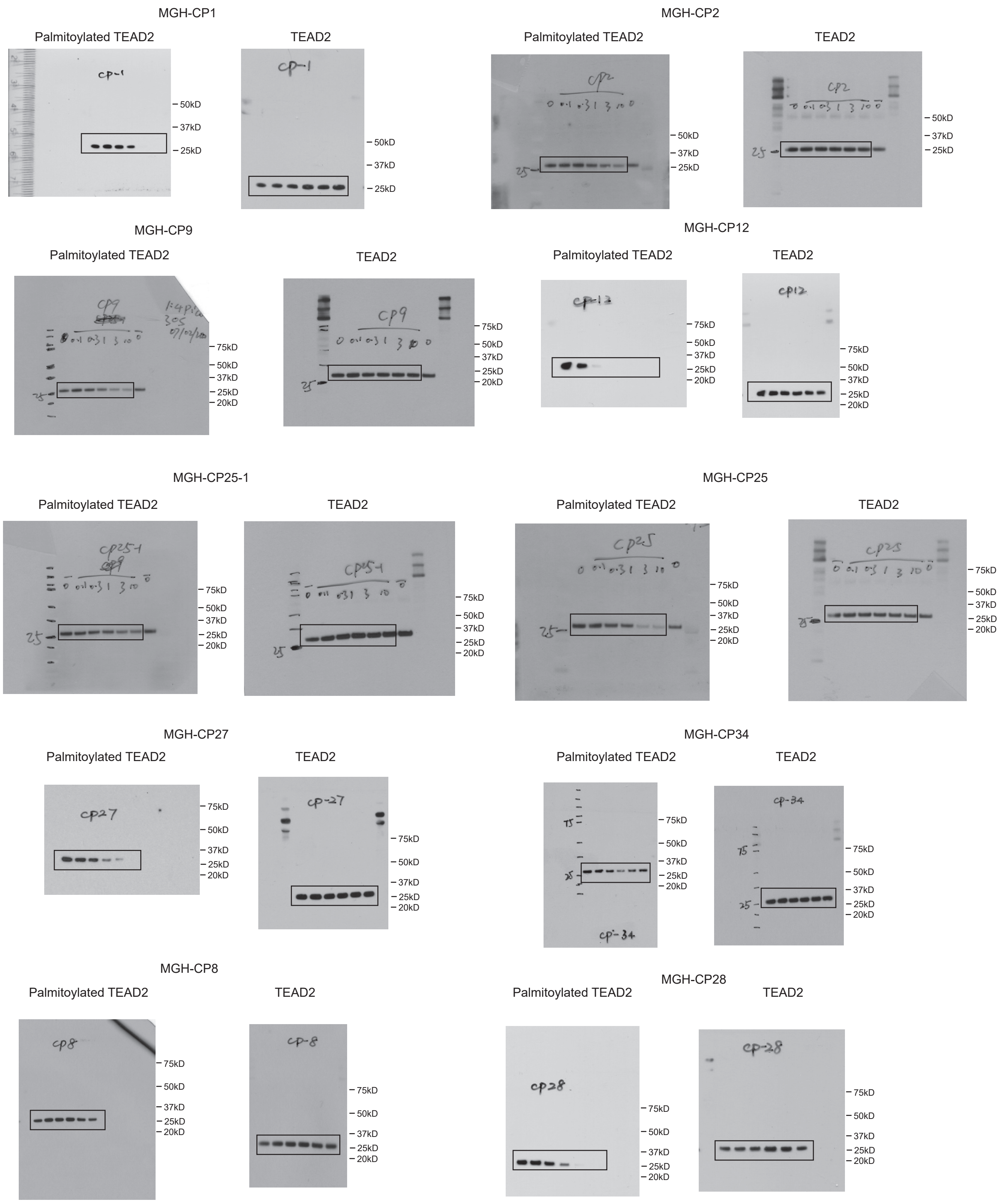

Supplementary Figure 14. Uncropped gel images for Supplementary Figure 1a.

Supplementary Figure 1b

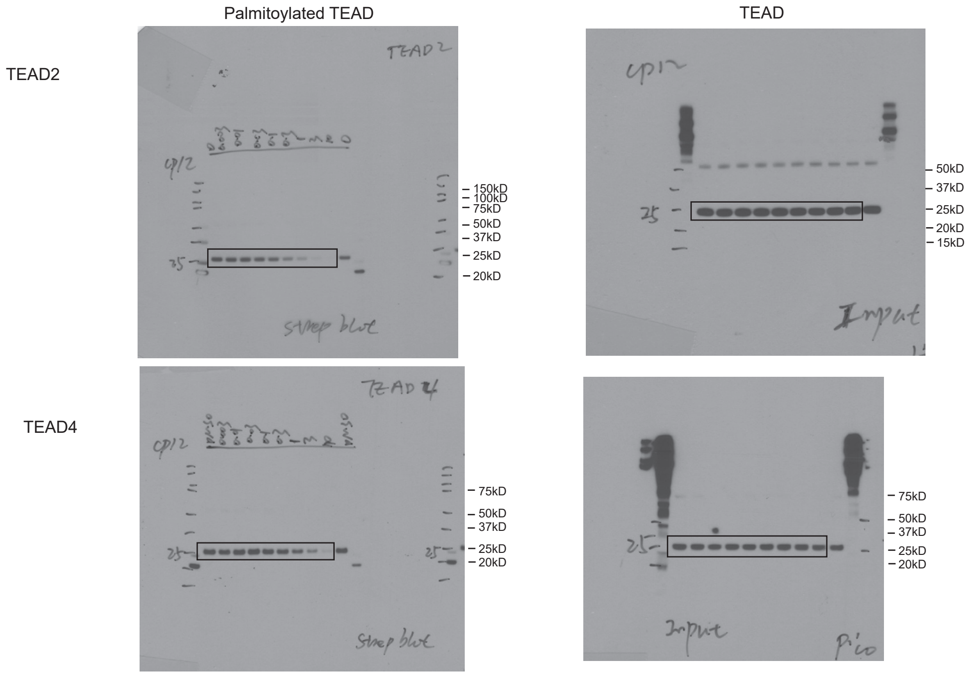

Supplementary Figure 1c

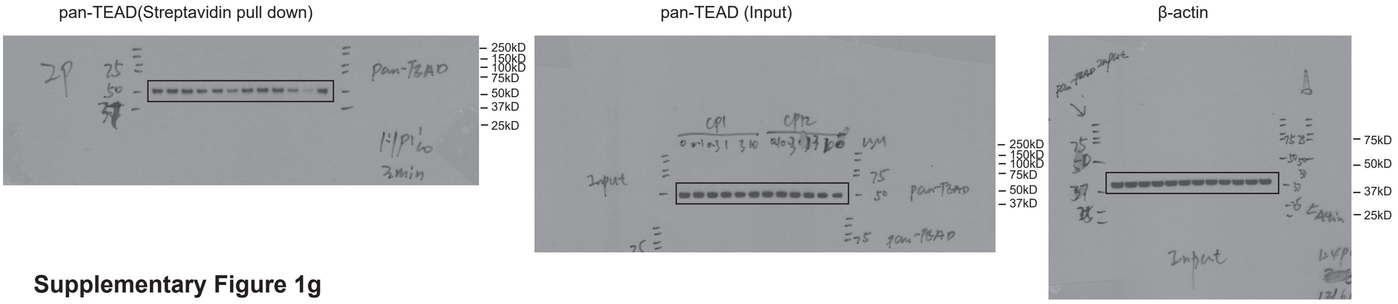

Supplementary Figure 1g

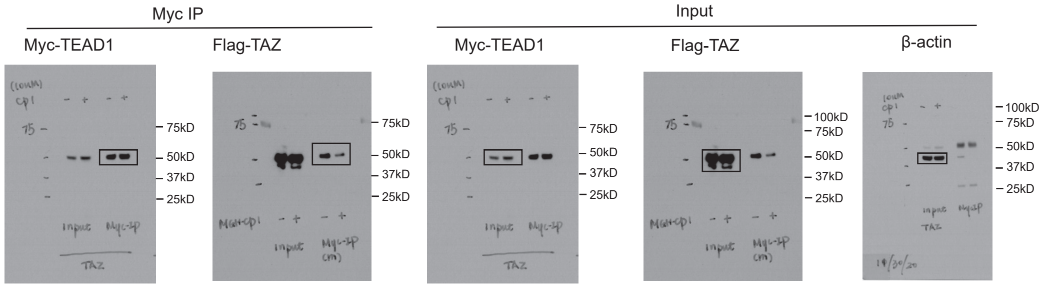

Supplementary Figure 1h

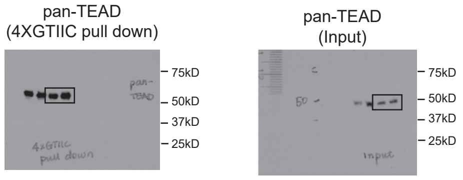

Supplementary Figure 15. Uncropped gel images for Supplementary Figure 1b, 1c, 1g and 1h.

a

Cell Cycle

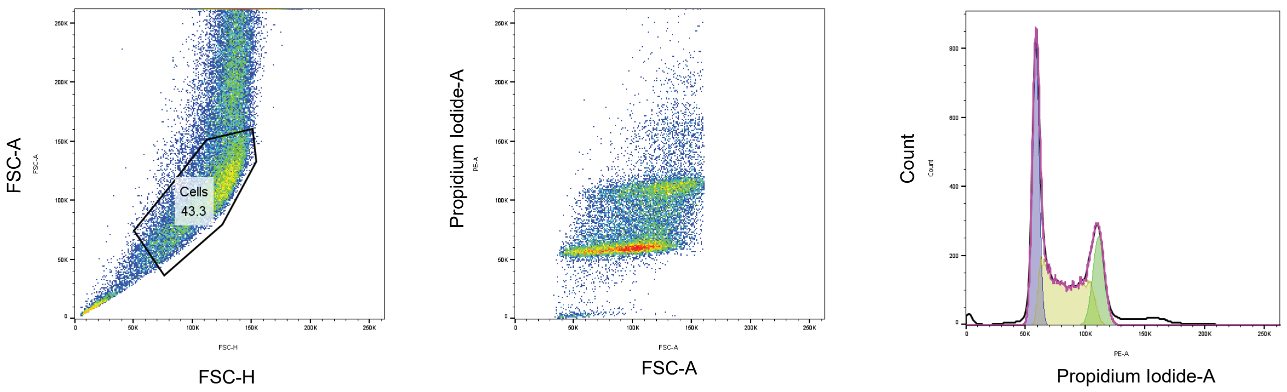

b

Cell Death

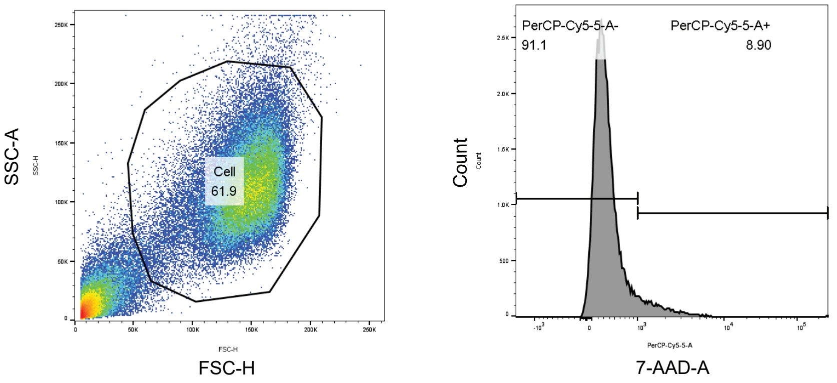

Supplementary Figure 16. Flow Cytometry Gating Strategy
a. Flow Cytometry Gating Strategy for cell cycle analysis. **b.** Flow Cytometry Gating Strategy for cell death analysis.

Supplementary Table 1. List of commonly upregulated genes upon TEAD-YAP/TAZ blockade.

Commonly upregulated genes once YAP/TAZ-TEAD blocked					
OAS1	MB	BHLHE41	MYO18A	IRF1	SCARB2
SOX4	RARRES3	KCNN4	BMP1	GRN	
GPRC5C	ARRB1	TSC22D1	ABCA1	CD74	
GBP2	AKR1C3	TSC22D4	PSAP	ATXN1	
PIK3C2B	BTN3A3	ACAP3	ZCCHC24	ZFAND5	
ST3GAL5	TP53INP1	DSC2	SELM	NRP1	
COL6A1	GABARAPL1	LRP1	KLHDC8B	TMEM87B	
S100A6	DDR1	ARRDC3	KIAA1147	SMPD1	
NR4A2	KDM6B	HBP1	ERAP2	TAPBP	
CTSD	TNFSF10	CLK4	G6PD	ATP6AP1	

Supplementary Table 2. Reagent List

Reagent list

REAGENT or RESOURCE	SOURCE	IDENTIFIER	Clone#	Dilution
**Antibody**				
Myc-tag	E M D Millipore	Cat#MABE282	9E10	1:1,000
Myc-Tag	Cell Signaling	Cat#2278S	71D10	1:1,000
Flag-tag	Cell Signaling	Cat#2368S		1:1,000
Flag-tag	Sigma	Cat#F1804	M2	1:1,000
HA-tag	Cell Signaling	Cat#3724S	C29F4	1:1,000
His-Tag	Invitrogen	Cat#MA1-21315	HIS.H8	1:10,000
β-actin	ABCAM	Cat#ab6276	AC-15	1:5,000
Streptavidin-HRP	Life Technologies	Cat#S911		1:5,000
Ki67	Cell Signaling	Cat#9027S		1:500
Phospho Histone 3	Abcam	Cat#ab5176	D2H10	1:500
YAP/TAZ	Cell Signaling	Cat#8418S	D24E4	1:1,000
p-AKT (S473)	Cell Signaling	Cat#4060S	clone D9E	1:1,000
p-AKT(T308)	Cell Signaling	Cat#9275S		1:1,000
AKT	Cell Signaling	Cat#2920S	40D4	1:1,000
Anti-Rabbit HRP	Cell Signaling	Cat#7074S		1:5,000
Anti-Mouse HRP	Cell Signaling	Cat#7076S		1:5,000
Alexa Fluor-488 secondary antibody	Invitrogen	Cat#R37118		1:500
**Plasmid**				
PRK5-mycTEAD1	Addgene	Cat#33109		
pcDNA3.1 myc-TEAD4	In house made			
pcDNA3.1 Flag-YAP1	Addgene	Cat#18881		

8XGTIIC-Luc	Addgene	Cat#34615
pGL2-Gal4-UAS-Luc	Addgene	Cat#33020
pCMX- Gal4-TEAD1	Addgene	Cat#33108
pCMV-Gal4-TEAD2	Addgene	Cat#33107
pcDNA-HA-VGLL4	In house made	
Tet-PLOK-puro	Addgene	Cat#21915
pcDNA-VGLL1-Flag	Genscript	Cat# OHu19565D
pcDNA-VGLL2-Flag	Genscript	Cat#OHu09252D
pcDNA-VGLL3-Flag	Genscript	Cat#OHu09259D
pcDNA-VGLL4-Flag	Genscript	Cat# OHu15805D
pWPXL	Addgene	Cat# 12257
pWPXL-SOX4	Addgene	Cat# 36984
pLEX303GFP	Addgene	Cat# 162032
pLEX303GFP-PIK3C2B	Addgene	Cat# 162001
**Reagent**		
Tris(2-carboxyethyl) phosphine hydrochloride (TCEP**)**	Sigma-Aldrich	Cat# C4706
Copper(II) sulfate	Sigma-Aldrich	Cat#496130
Tris[(1-benzyl-1*H*-1,2,3-triazol-4-yl)methyl]amide (TBTA)	Sigma-Aldrich	Cat# 678937
MES hydrate	Sigma-Aldrich	Cat# 76039-100mL-F
Alkyne Palmitoyl-CoA (trifluoroaetate salt)	Cayman	Cat#15968
Biotin Picolyl Azide	Click Chemistry Tools	Cat#1167-5
Alkynyl Palmitic acid	Click Chemistry Tools	Cat#1165

Streptavidin Agarose beads	Life Technologies	Cat# SA10004
Protein A/G XPure Agarose Resin	UBPBio	Cat#P5030-1
Anti-FLAG® M2 Magnetic Beads	Sigma-Aldrich	Cat#M8823
Laemmli (SDS-Sample Buffer, Reducing, 6X)	Boston BioProducts	Cat#BP-111R
cOmplete EDTA-free protease inhibitors cocktail	Roche	Cat#05892791001
Phosphatase inhibitor cocktail	Sigma-Aldrich	Cat#P0044
Trizol reagent	Invitrogen	Cat#15596026
Calcein-AM	Biolegend	Cat#425201
Propidium Iodide Solution	Biolegend	Cat#421301
7-AAD Viability Staining Solution	Biolegend	Cat# 420403
Protein Western Blotting SuperSignal West Pico Plus Chemiluminescent Substrate	Life Technologies	Cat# 34580
Immobilon Forte Western HRP substrate	Sigma	Cat# WBLUF0500
SeaPrep agarose	Lonza	Cat#50302
DPBS (Gibco#10010)	Life Technologies	Cat#14190250
VGLL3 siRNA	HORIZON DISCOVERY	Cat#M-031975-02-0010
PIK3C2B siRNA pool	HORIZON DISCOVERY	Cat#M-006772-01-0010
SOX4 siRNA-1	Sigma	SASI_Hs01_00188751
SOX4 siRNA-1	Sigma	SASI_Hs01_00188752
siRNA Universal Negative Control #1	Sigma	Cat#SIC001

Supplementary Table 3. Primer list

The following primers were used in Quantitative RT-PCR:

hCyr61	Forward	GGAAAAGGCAGCTCACTGAAGC
	Reverse	GGAGATACCAGTTCCACAGGTC
hCTGF	Forward	CTTGCGAAGCTGACCTGGAAGA
	Reverse	CCGTCGGTACATACTCCACAGA
hANKRD1	Forward	CGACTCCTGATTATGTATGGCGC
	Reverse	GCTTTGGTTCCATTCTGCCAGTG
hGAPDH	Forward	GTCTCCTCTGACTTCAACAGCG
	Reverse	ACCACCCTGTTGCTGTAGCCAA
hβ-actin	Forward	CACCATTGGCAATGAGCGGTTC
	Reverse	AGGTCTTTGCGGATGTCCACGT
hYAP1	Forward	TGTCCCAGATGAACGTCACAGC
	Reverse	TGGTGGCTGTTTCACTGGAGCA
hTAZ	Forward	ACCGTGTCCAATCACCAGTCCT
	Reverse	CCTTGGTGAAGCAGATGTCTGC
hTEAD1	Forward	CCTGGCTATCTATCCACCATGTG
	Reverse	TTCTGGTCCTCGTCTTGCCTGT
hTEAD2	Forward	CCGCTACATCAAGCTGAGAACG
	Reverse	GGTTGCCATTGTCTGGAAAGCC
hTEAD3	Forward	AGGCAGTAGATGTGCGCCAGAT
	Reverse	TCCTGGATGGTGCTGTTGAGGT
hTEAD4	Forward	GAAGGTCTGCTCTTTCGGCAAG
	Reverse	GAGGTGCTTGAGCTTGTGGATG
hPIK3C2B	Forward	CCTCCTGAAACGAGCTGTGTCT
	Reverse	CACAGTAAGGCTGCCAGCAGAT
hSOX4	Forward	GACATGCACAACGCCGAGATCT
	Reverse	GTAGTCAGCCATGTGCTTGAGG
